# Supplementary material for: Integrative construction of regulatory region networks in 127 human reference epigenomes by matrix factorization
Source: Nucleic Acids Res. 2019 Jul 2;47(14):7235–46. doi: 10.1093/nar/gkz538 (PMC6698807; doi:10.1093/nar/gkz538)
Supplement: gkz538_Supplemental_File [file gkz538_supplemental_file.docx]

**Supplementary Materials and Methods**

**Different sizes of sliding window**

Different sizes of slide windows were explored. The AUCROC of SWIPE-NMF predictions using ChiA-PET as gold standard and the running time for each sliding window using window sizes 2, 5,10,20 M bp is shown in S Table 2. The analysis was conducted on a randomly selected 20 M bp region on Chr 10 of K562 cell (20,000,000 to 40,000,000). The running time of a single window was tested on a computer with 2.8 GHz Intel Core i7 CPU.

**S Table 1 Data used as experimental sources of evidence for potential distal regulatory interactions.**

| **Data type** | **Description** | **Evidence** | **References** |
| --- | --- | --- | --- |
| Enhancer-promoter coactivity | Coactivity is inferred by activity correlation between enhancer and promoters using histone enrichment signal tracks and the expression level of promoter. | Similar to the inference of gene functional associations by means of co-expression analysis, it has been proposed that covariation of epigenomic activity profiles between regions (in this case cis-regulatory elements and promoters) both across and within tissues is indicative of functional associations. | [(Ernst et al. 2011; Roadmap Epigenomics Consortium et al. 2015; Marstrand & Storey 2014)](https://paperpile.com/c/mRaETQ/0p8v+DsDi+xYF3). |
| Hi-C (PCHi-C) | It is the genome-wide version of the original chromatin conformation technique (3C): uses high-throughput sequencing to quantify proximity ligation products in contact libraries. Significant pairwise physical interactions between loci are usually called from contact matrices.  Further enrichment for interactions of interest can be achieved by sequence capturing methods. PCHi-C, was proposed to pull down only those fragments containing promoters and their interacting regions from Hi-C libraries. | Hi-C is often used as the reference source of physical evidence of interaction between loci, irrespective of distance. By mapping enhancers and promoters to interacting loci, potential enhancer-promoter interactions, supported by evidence physical 3D spatial proximity, can be proposed. | [(Fraser et al. 2015; Schmitt et al. 2016; Lieberman-Aiden et al. 2009; Javierre et al. 2016)](https://paperpile.com/c/mRaETQ/rcZI+6IZI+yiR1+lATy). |
| Expression quantitative trait loci (eQTL) | Expression quantitative trait loci (eQTL) are genomic loci that contribute to variation in RNA expression levels. | Mapped eQTLs are often considered to imply tissue-specific functional associations between cys-regulatory elements and target genes. | [(GTEx Consortium 2015)](https://paperpile.com/c/mRaETQ/z0Cl). |
| Topologically associating domains (TADs) | In addition to enhancer-promoter interactions, higher-order organizational domains can also be statistically defined from Hi-C data. TADs are blocks of dense chromatin which interact more frequently within themselves than with neighboring regions. | It has been documented that most long-range regulatory interactions between enhancers and genes is constrained within TADs. Thus TAD annotations provide a reference for the expectation of the localization and length range of enhancer-promoter interactions. | [(Fraser et al. 2015; Schmitt et al. 2016; Jost et al. 2017)](https://paperpile.com/c/mRaETQ/rcZI+6IZI+Icia). |
| DNase I hypersensitivity sites (DHSs) | DNase I hypersensitive sites (DHSs) are markers of regulatory activity associated with cis-regulatory elements that define regions of chromatin accesibility. | It has been documented that active enhancers become synchronously hypersensitive to DNaseI with their target promoter. Thus covariation patterns of accessibility between enhancers and promoters provide evidence suggestive of functional association. | [(Thurman et al. 2012;](https://paperpile.com/c/mRaETQ/OHm3) [Roadmap Epigenomics Consortium et al. 2015](https://paperpile.com/c/mRaETQ/0p8v+DsDi+xYF3)[)](https://paperpile.com/c/mRaETQ/OHm3). |

Table S2 Performance and running time of SWIPE-NMF on a randomly picked 20M bp region using different window sizes.

| Window size | 2 M bp | 5 M bp | 10 M bp | 20 M bp | >20M bp |
| --- | --- | --- | --- | --- | --- |
| AUCROC | 0.83 | 0.85 | 0.86 | 0.84 | NA |
| Running time for one window | ~10 mins | ~15 mins | ~1 hr | ~8 hrs | Not computationally feasible |

Table S3 Data source accession number or URL

| Data set | Accession number or URL |
| --- | --- |
| Schmitt, A.D., Hu, M., Jung, I., Xu, Z., Qiu, Y., Tan, C.L., Li, Y., Lin, S., Lin, Y., Barr, C.L. *et al.* (2016) | GEO: GSE87112.­­ |
| Rao, S.S.P., Huntley, M.H., Durand, N.C., Stamenova, E.K., Bochkov, I.D., Robinson, J.T., Sanborn, A.L., Machol, I., Omer, A.D., Lander, E.S. *et al.* (2014) | GSE63525 |
| Thurman, R.E., Rynes, E., Humbert, R., Vierstra, J., Maurano, M.T., Haugen, E., Sheffield, N.C., Stergachis, A.B., Wang, H., Vernot, B. *et al.* (2012) | GSE29692, GSE32970 |
| Dixon, J.R., Selvaraj, S., Yue, F., Kim, A., Li, Y., Shen, Y., Hu, M., Liu, J.S. and Ren, B. (2012) Topological domains in mammalian genomes identified by analysis of chromatin interactions. *Nature*, **485**, 376-380. | GSE35156 |

**Supplementary Figure 1**. Performance of SWIPE-NMF using ChiA-PET as gold standard on two randomly picked 5M bp window in K516 cell.
